# HYPHAEdelity: a quantitative image analysis tool for assessing peripheral whole colony filamentation

**DOI:** 10.1093/femsyr/foac060

**Published:** 2022-11-18

**Authors:** Scott J Britton, Lisa J Rogers, Jane S White, Dawn L Maskell

**Affiliations:** Institute of Biological Chemistry, Biophysics, and Bioengineering, School of Engineering and Physical Sciences, Heriot-Watt University, Edinburgh, United Kingdom EH14 4AS; TranscendED, Brooklyn, NY 11232, United States; Institute of Biological Chemistry, Biophysics, and Bioengineering, School of Engineering and Physical Sciences, Heriot-Watt University, Edinburgh, United Kingdom EH14 4AS; Institute of Biological Chemistry, Biophysics, and Bioengineering, School of Engineering and Physical Sciences, Heriot-Watt University, Edinburgh, United Kingdom EH14 4AS

**Keywords:** phenotypic switching, filamentous growth, pseudohyphal differentiation, morphogenesis, whole colony morphology, image analysis, yeast

## Abstract

The yeast *Saccharomyces cerevisiae*, also known as brewer's yeast, can undergo a reversible stress-responsive transition from individual ellipsoidal cells to chains of elongated cells in response to nitrogen- or carbon starvation. Whole colony morphology is frequently used to evaluate phenotypic switching response; however, quantifying two-dimensional top-down images requires each pixel to be characterized as belonging to the colony or background. While feasible for a small number of colonies, this labor-intensive assessment process is impracticable for larger datasets. The software tool HYPHAEdelity has been developed to semi-automate the assessment of two-dimensional whole colony images and quantify the magnitude of peripheral whole colony yeast filamentation using image analysis tools intrinsic to the OpenCV Python library. The software application functions by determining the total area of filamentous growth, referred to as the *f-measure*, by subtracting the area of the inner colony boundary from the outer-boundary area associated with hyphal projections. The HYPHAEdelity application was validated against automated and manually pixel-counted two-dimensional top-down images of *S. cerevisiae* colonies exhibiting varying degrees of filamentation. HYPHAEdelity's *f-measure* results were comparable to areas determined through a manual pixel enumeration method and found to be more accurate than other whole colony filamentation software solutions.

## Teaser

A novel quantitative image analysis tool for the assessment of peripheral whole colony filamentation in *Saccharomyces cerevisiae*.

## Introduction

*Saccharomyces cerevisiae*, an industrially relevant budding yeast, can undergo a reversible stress-responsive transition from individual ellipsoidal cells (yeast form) to filaments of thinly elongated cells (filamentous form) in response to nitrogen- or carbon starvation (Gimeno et al. 1992 , Mösch and Fink 1997, Lengeler et al. 2000, Mösch 2000, Ceccato-Antonini and Sudbery 2004, Etschmann and Schrader 2006, Lodolo et al. 2008, Britton et al. 2021, Wauters et al. 2021). This phenotypic switch allows microorganisms to undergo rapid microevolution to adapt to constantly changing microenvironments (Jain and Hasan 2018). This transition is often viewed as an analogous adaptive response to motility in sessile cells, where the reorganization of polarity, increase in cell length, and incomplete scission permits the broader exploration of the environment and facilitates the foraging for available nutrients under nutrient-limiting conditions (Gimeno et al. 1992, Kron 1997, Gancedo 2001, Hornby et al. 2001, Chen et al. 2004, Nickerson et al. 2006, Cullen and Sprague 2012). Under these conditions, cells cultured on agar will radiate outward from colony centres across surfaces (diploid) or downward into the agar surface (haploid), depending on their ploidy (Chow et al. 2019). However, the inherent genetic machinery employed for diploid pseudohyphal growth and invasive haploid growth differs only marginally (Cullen and Sprague 2000, Chen and Fink 2006, Zaman et al. 2008, Cullen and Sprague 2012).

Five major evolutionarily conserved signaling pathways have thus far been identified to mediate nutrient-induced developmental responses in yeast: (i) the cAMP-PKA pathway; (ii) the TOR pathway; (iii) the SNF1/AMPK pathway; (iv) the Rim101 pathway; and (v) the Kss1-MAPK pathway (Roberts and Fink 1994, Ceccato-Antonini 2008, Granek et al. 2011, Cullen and Sprague 2012, Ryan et al. 2012). These pleiotropic signaling pathways play a role in the transcriptional expression of *FLO11*, previously designated as *MUC1*, encoding a cell surface flocculin protein with a structure comparable to yeast serine/threonine-rich glycosylphosphatidylinositol(GPI)-anchored cell wall proteins (Lo and Dranginis 1998, Zara et al. 2009). Beyond filamentous growth, these specialized cell-surface flocculins are additionally known for their involvement in surface adhesion; biofilm/mat formation; velum development; and flocculation, an asexual calcium-dependent form of cell-cell aggregation (Lo and Dranginis 1998, Bayly et al. 2005, Fidalgo et al. 2006, Zara et al. 2009, Soares 2011, Andersen 2014, Yang et al. 2018, Chow et al. 2019, Bouyx et al. 2021, Huismann et al. 2021).

Adaptive phenotypic switching responses are not unique to *S. cerevisiae*, but are a widespread phenomenon commonly observed across many different fungi, including the fungal pathogens *Candida albicans*, *Candida tropicalis*, *Candida glabrata*, *Candida lusitaniae*, *Candida auris*, *Cryptococcus neoformans*, and *Trichosporon* spp. (Wickes et al. 1996, Goldman et al. 1998, Csank and Haynes 2000, Lengeler et al. 2000, Vargas et al. 2000, D'Souza and Heitman 2001, Lachke et al. 2002, Jain et al. 2006, Pu et al. 2014, Mohammadshirazi and Kalhor 2016, Perini et al. 2019, Dunn et al. 2020, Bouyx et al. 2021, Momani et al. 2021, Fan et al. 2021). These pathogens can be particularly harmful to immunocompromised individuals, like those with neutropenia, hematopoietic stem cell or solid organ transplantation, high-dose corticosteroid treatment, HIV infection, or immune suppression resulting from anti-cancer and cytotoxic therapies (Filler et al. 2005, Ben-Ami et al. 2008, Mousset et al. 2014).

Fungi invade the milieu by generating hyphal filaments and extracellular hydrolases, where the yeast-to-filament transition plays a significant role in host–cell attachment, facilitating tissue invasion, damage, and evasion from host defenses (Rooney and Klein 2002). In pathogenic fungi, the switch from budding to filamentous growth is a dimorphic transition often related to virulence, and some investigations have demonstrated that the mechanical forces associated with hyphal growth in some fungi were sufficient to cause cell damage and enable penetration into epithelial cells even in the absence of secreted virulence factors (Moyes et al. 2016, Westman et al. 2019). Therefore, the transition between yeast and filamentous forms has been a primary focus of contemporary fungal virulence research due to their significant cause of mortality in humans worldwide.

However, as insufficient genomic and biochemical resources exist for many pathogenic organisms demonstrating phenotypic switching, *S. cerevisiae* is often exploited as a model organism for these investigations due to its genetic tractability and well-documented biochemical characterization (Botstein and Fink 2011, Cullen and Sprague 2012). Further, as some of the key *S. cerevisiae* signaling pathways involved in the control filamentous growth are highly conserved in fungal pathogens, like *C. albicans* and other more distantly related fungi, *S. cerevisiae* has become an attractive model organism for investigating complex fungal morphologies, the genetic contributions to virulence in fungal pathogens, or eukaryotic cell differentiation in response to extrinsic fungal cues (Cullen and Sprague 2012, Ryan et al. 2012).

A standard technique to quantify filamentous growth involves capturing a two-dimensional top-down digital whole colony image, and then classifying each pixel as belonging to the annular mass, a filament protuberance, or the background (Gimeno et al. 1992 , Lorenz et al. 2000, Ruusuvuori et al. 2014, Binder et al. 2015, Tronnolone et al. 2017). This measurement approach utilizes the relative pixel areas between the annular mass and the protruding filaments as a metric for ascertaining the filamentation degree. Although possible to execute the classification of each pixel manually, this undertaking would be impractical and strenuous for studies containing a significant number of experimental images. For example, high-throughput assays originating from genome-wide mutant libraries are regularly exploited to establish the link between specific genes, variation in growth patterns, and virulence (Ryan et al. 2012). Such studies often yield a significant number of experimental images, and require robust methods, for identifying and quantifying alterations in morphology.

Therefore, we aimed to develop HYPHAEdelity, a publicly available and user-friendly image analysis software tool to facilitate the semi-automated quantification of peripheral whole colony filamentation. The HYPHAEdelity application allows for the importation, automated conversion of whole colony images to grayscale, and accurate calculation of filamentation degree, referred to here as the *f-measure*, without advanced knowledge of image analysis, image processing, or computer programming. Furthermore, once downloaded, HYPHAEdelity can be installed and operable in less than five minutes whilst not requiring the installation of additional plugins or modification to standard system configurations. HYPHAEdelity is publicly available at github.com/unstablelimitcycle/hyphaeDetection.

## Materials

### Strains

Two strains of *Saccharomyces cerevisiae*, YMD4527, and YMD4544, were obtained from the yeast collections of Duvel Moortgat, NV (Puurs-Sint-Amands, BE). Per manufacturer instructions, taxonomic identities were verified by real-time multiplex PCR (GEN-IAL, Troisdorf, Germany, Cat. No. Q072).

### Media

Yeast strains were pre-cultured in a liquid broth [YPD; 1% yeast extract, 2% peptone, and 2% dextrose]. Filamentous growth was induced on 4X synthetic low-ammonium dextrose agar [SLAD agar; 0.68% yeast nitrogen base w/o amino acids or ammonium sulfate, 2% dextrose (Biowest, Nuaillé, FR), 50 µM ammonium sulfate, and 2% washed agar (VWR International, Leicestershire, UK, Cat. No. 20 767)] and where applicable, supplemented with 100 µM 2-phenylethanol (ThermoFisher Scientific, Perth, UK, Cat. No. A15241). Unless otherwise indicated, all materials were obtained from Sigma Aldrich (Ayr, UK).

### Filamentous growth assay

*Saccharomyces cerevisiae* strains were pre-cultured in YPD at 24°C + 2°C for 24 h. Cultures underwent serial dilution in 0.85% sterile saline and were inoculated to a density of 1.0 × 10^2^ on SLAD agar plates. For some experimental conditions, SLAD was supplemented with 100 µM exogenous 2-PE. Plates were sealed with parafilm and incubated inverted for three days at 30°C. Following incubation, colonies were randomly selected and photographed from each dish at 2.5 X total magnification using a ZEISS Axio Lab.A1 FL-LED microscope equipped with a ZEISS Axiocam 105 colour camera (2.2 μm pixel resolution) and saved as a JPEG image. The assay was carried out in triplicate per condition, whereafter, eight to ten whole colony photographs from each assay condition (n = 30) were chosen for further image analysis and processing.

## Image Analysis and Processing

Python's OpenCV (www.opencv.org) open-source library algorithms for smoothing noise, thresholding, finding contours, and identifying/counting pixels were used to analyze two-dimensional images of singular whole yeast colonies uploaded into HYPHAEdelity (Brooklyn, USA). Upon uploading JPEG images, HYPHAEdelity initializes a series of arrays for data storage and sequentially initiates an automated six-step analysis cycle (S1-S6) for each uploaded image. In S1, images of the allowed file type (JPEG) are uploaded from a given folder at a uniform resolution (2560 × 1920), separated from any attached supplementary metadata, and a designated pathway is created to read and write the new files. Uploaded images must exceed a minimum resolution of 96 pixels x 96 pixels to guarantee consistent and accurate whole colony assessment. It is also recommended that whole colony images be minimally captured under 2.5 X total magnification. Here the file name designations for all images are stored for future review and visualization. In S2, the uniform resolution JPEG images are automatically converted from blue-green-red (BGR) to grayscale using OpenCV's cvtColor function. When smoothing images in OpenCV, a box filter is the default filter type; however, due to the linear nature of a box filter, which equally weights all samples within a square region of the image, the highest frequency values of a given image would be treated with the same filter intensity as the lower frequency values. This does not capture the detail of filamentation needed for the accurate measuring of hyphae growth. Hence, a Gaussian blur filter with a 5 × 5 rectangular kernel sufficiently smoothed background noise while also preserving the extent of the filaments projected by the yeast colonies (S3).

Post-Gaussian blurred images were subsequently converted to binary images, also known as image thresholding, (S4) by applying a simple manual threshold. While adaptive thresholding techniques are often recommended for large volumes of images, a manually set threshold works best in this situation since all of the images used are of similar external morphology and were taken all under homogeneous lighting and environmental conditions. In future versions of the program, we will add the option to choose between adaptive and manual thresholds to account for varying input image type and quality. We initially used Otsu's method (Otsu 1979) of adaptive thresholding for the first iteration of the HYPHAEdelity software, but switched to a manually-set threshold when we discovered that Otsu's method did not capture the granularity of more noisy images. Otsu's method is an adaptive global thresholding method that looks for a threshold that minimizes intraclass variance, and as a result works the most efficiently with bimodal distributions of data.

**Figure 1.**
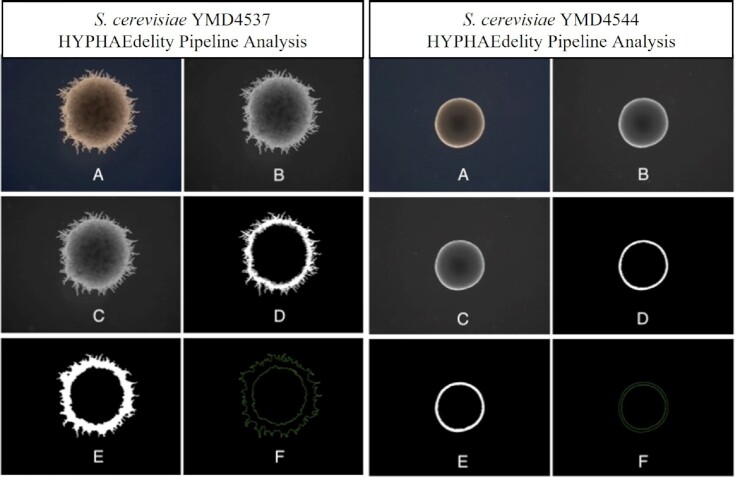
Example of image processing pipeline for filamentous *S. cerevisiae* strain YMD4537 (Left) and non-filamentous strain *S. cerevisiae* strain YMD4544 (Right) captured under a total magnification of 2.5x. Panels demonstrate the following steps within the processing pipeline: (**A**) Original Image (**B**) Greyscale Image (**C**) Gaussian Blurred Image (**D**) Thresholded Image (**E**) Closed Image (**F**) Contoured Image.

Next, discontinuous objects were morphologically closed by employing a 5 × 5 rectangular kernel to eliminate small voids (S5) caused by multiple filaments overlapping or returning to the original colony. In the final step (S6), OpenCV's findContours() function detects all contours within the thresholded and closed images. The two largest contours present in each image account for (i) the boundary of all of the filamentous protrusions outside the central colony mass (referred to as A*_outer_*) and (ii) the boundary of the central colony mass (referred to as A*_inner_*). The total filamentous growth area is computed by subtracting the smaller measured area from the larger measured area. The normalized growth index value, referred to here as the f-measure, is effectively the % change of the outer boundary area compared to the inner boundary area (Fig. 1).

$$\begin{eqnarray*}
{\rm f - measure} = \frac{{\left( {{A}_{outer} - {A}_{inner}} \right)}}{{{A}_{inner}}}\,*\,100
\end{eqnarray*}$$

HYPHAEdelity outputs pictures for all binarized, thresholded colonies; their contours; and a .csv file including original colony areas, filamentous growth areas, and growth indices with statistical measures of spread and center.

## Software Validation Results

The HYPHAEdelity two-dimensional peripheral whole colony analysis algorithm was validated against (i) six geometric binarized test images to determine the precision of the pixel counting function, (ii) an existing set of manually counted whole colony images to determine its accuracy compared to the traditional manual enumeration, and (iii) the existing whole colony measurement tool TAMMiCol (**T**ool for **A**nalysis of the **M**orphology of **M**icrob**i**al **Col**onies).

### Validation—Pixel counting accuracy

OpenCV's pixel counting accuracy with HYPHAEdelity was validated against six geometric binarized test images: two circles of different radii, two squares of different side lengths, and two triangles of different base lengths and the same height. The calculated areas of the geometric test figures, having basic geometric area formulas, were evaluated against the areas determined by OpenCV's findArea() function. The difference between the computed pixel and algorithmic areas determined by OpenCV's findArea() function was less than 1% for each geometric figure (See Table 1).

**Table 1.** Calculated areas of the geometric figures compared against areas determined by HYPHAEdelity OpenCV's findArea() function. The % difference observed across all geometric test images was ≤ 0.40%.

Geometric Image	HYPHAEdelity Calculated Area (px)	Computational Calculated Area (px)	Determined Area Difference (px)	% Difference
Circle (r = 500 px)	195,671	196,350	−679	0.16
Circle (r = 1000 px)	784,154	785,398	−1,244	0.20
Square (l = 500 px)	249,001	250,000	−999	0.25
Square (l = 1000 px)	998,001	1,000,000	−1,999	0.35
Triangle (b = 500 px)	80,595	81,400	−441	0.40
Triangle (b = 1000 px)	323,565	324,375	−810	0.25

The HYPHAEdelity algorithm and TAMMiCol software tool were evaluated against the AWRI 796 Sample 5 data set of manual pixel-counted (MPC) *S. cerevisiae* whole colony images produced by Binder et al. (2015). As seen in Table 2, the comparison between HYPHAEdelity and the MPC AWRI 796 Sample 5 dataset revealed an average difference of 1.11% and a maximum difference of 2.45% across all time points. In general, the competitive software TAMMiCol was comparatively less accurate than HYPHAEdelity and demonstrated a higher average difference (4.88%) and a maximum difference (13.62%) compared to MPC-determined values.

**Table 2.** HYPHAEdelity (H) evaluation against TAMMiCol (T) software and Manual Pixel Counting (MPC): Calculated area and % differences between the binary images produced by HYPHAEdelity and competitive software program TAMMiCol. In addition, the calculated binary images produced by TAMMiCol and HYPHAEdelity are compared further to the AWRI 796 Sample 5 manual pixel counted data set produced by Binder et al. 2015.

AWRI 796 Sample 5 (hrs)	HYPHAEdelity (H) Calculated Area (px)	Manual Pixel Counted (MPC) Area (px)	TAMMiCol (T) Pixel Counted Area (px)	% Difference (H/MPC)	% Difference (H/T)	% Difference, (T/MPC)
73	180,504	183,579	192,563	1.68	6.26	4.89
87	212,612	215,748	245,138	1.45	13.27	13.62
115	334,667	343,083	345,565	2.45	3.15	0.72
162	429,754	433,260	443,261	0.81	3.05	2.31
211	463,021	462,044	477,308	0.21	2.99	3.30
233	463,296	463,538	484,187	0.05	4.31	4.45
				x̄ = 1.11	x̄ = 5.51	x̄ = 4.88

### Validation—Pixel assignment

The algorithm was then evaluated against a novel set of 77 whole colony images obtained from a 3-day incubation at 28°C of a filamentous (YMD4537) and a non-filamentous (YMD4544) strain of *S. cerevisiae* cultured on SLAD medium. YMD4537 was also simultaneously cultured on SLAD medium supplemented with 100 µM 2-PE to stimulate a dynamic range of filament production (Chen and Fink 2006). Here, the assignment of pixels associated with the annual centres of each whole colony image was compared between HYPHAEdelity, TAMMiCol, and manual pixel counting. All software analysis was performed according to developer instructions.

Compared to the manually processed images, the HYPHAEdelity-determined annular counts were more comparable than those determined by the pre-existing whole colony analysis software tool TAMMiCol. As seen in Table 3, the pairwise mean % difference, associated with the annular area determination, between the HYPHAEdelity software and manual pixel counting was marginal, ranging from 0.56 ± 0.13 to 0.64 ± 0.15 between the three experimental conditions spanning a dynamic range of *f-measures* between 0.27 ± 0.08 and 0.71 ± 0.38. Conversely, between experimental conditions, the pairwise mean % difference between TAMMiCol and manual pixel counting ranged from 5.09 ± 0.60 to 5.12 ± 0.56. Similar differences were also observed between TAMMiCol and HYPHAEdelity.

**Table 3.** Calculated pairwise mean % difference between YMD4537 (0 µM 2-PE), YMD4537 (100 µM 2-PE), and YMD4544 (0 µM 2-PE) determined by HYPHAEdelity (H), TAMMiCol (T), and Manual Pixel Counting (MPC).

Strain	Condition	N	Mean *f-measures*	(H) vs. (MPC) Mean ± STD	(T) vs. (MPC) Mean ± STD	(H) vs. (T) Mean ± STD
YMD4537	0 µM 2-PE	30	0.53 ± 0.14	0.58 + 0.12%	5.01 ± 0.50%	5.82 ± 0.56%
YMD4537	100 µM 2-PE	25	0.71 ± 0.38	0.64 + 0.15%	5.12 ± 0.56%	5.76 ± 0.65%
YMD4544	0 µM 2-PE	22	0.27 ± 0.08	0.56 + 0.13%	5.09 ± 0.60%	5.98 ± 0.60%

## Discussion

Microorganisms, including pathogenic fungi, can participate in a myriad of social phenotypes that provide fitness advantages to individuals and the population (West et al. 2006). Lately, there is a growing awareness that microorganisms communicate and cooperate to accomplish many multicellular behaviors, such as biofilm formation, tissue invasion, and filamentous growth; this has become a subject of considerable interest as many of these behaviors are involved in establishing disease and fungal virulence.

The motivation to develop HYPHAEdelity, a publicly available automated analysis software tool for evaluating whole colony filamentous growth, was inspired largely by the initial studies demonstrating filamentation's importance in fungal disease establishment and virulence (Calera et al. 2000, Shapiro et al. 2011, Vila et al. 2017). As in some studies, the technique utilized to quantify filamentation degree involves capturing a two-dimensional top-down digital whole colony image, and then classifying each pixel as belonging to the annular mass, a filament protuberance, or the background, where the standard measurement approach utilizes the relative pixel areas between the annular mass and the protruding filaments. However, due to this analysis's strenuous and manual nature, this undertaking would be too laborious and impractical for investigations involving more extensive data collections, perhaps containing hundreds or thousands of experimental images. Consequently, the HYPHAEdelity software tool allows for the straightforward semi-automated quantification of *S. cerevisiae* peripheral whole colony filamentation, demonstrating a suitable degree of deviation compared to manual pixel area evaluation without the necessity for specialized user knowledge in computer programming or image analysis due to its easy to use graphical user interface.

A MacBook Pro laptop running OSX 11.1 with a 2.7 GHz Quad-Core Intel Core i7 processor takes approximately 20 seconds to transform high-resolution yeast colonies images to binary images, with identified edges and filamentous growth identified, and subsequently calculates the area and standardized *f-measure* of filamentous growth from the colony. The assessment of a single image manually could take 15 minutes, while on average, the analysis completed with HYPHAEdelity takes about 20 seconds per image to evaluate, which is also superior to other currently available software that may take several hours to analyze 10 images (Tronnolone et al. 2018).

Therefore, introducing a semi-automated two-dimensional whole colony image analysis software capable of accurately measuring peripheral filamentous growth in *S. cerevisiae* is an added value to those analyzing large whole colony data sets. It is envisioned that this would include investigations related to fungal pathogenesis, where whole colony analysis is an essential metric in identifying and characterizing strain-specific growth patterns, as well as the effectiveness of antifungals on inhibiting pseudohyphal formation, a key contributor to fungal virulence. Even so, the semi-automated software tool is not limited to only clinical applications, but in fact could easily be used to analyze the filamentous growth of yeast in other systems or further adapted to measure other whole colony traits such as sliding behavior with further development (Gori et al. 2011). However, as the software has been optimized and validated here only for use with *S. cerevisiae*, appropriate validation measures should be taken if users decide to employ this tool for use with different filamentous organisms.
